# The Perception of Surgery Program Directors of Residency Applicants From Longitudinal Integrated Curriculum Medical Schools: A Survey of the Association of Program Directors in Surgery Members

**DOI:** 10.7759/cureus.34375

**Published:** 2023-01-30

**Authors:** Rachel A Kracaw, Jessica Hernandez-Moreno, Oscar I Estrada Munoz, Corrin C Sullivan, Neil Haycocks, Gary K Shen

**Affiliations:** 1 Medical Education, Kirk Kerkorian School of Medicine at the University of Nevada, Las Vegas (UNLV), Las Vegas, USA; 2 General Surgery, Kirk Kerkorian School of Medicine at the University of Nevada, Las Vegas (UNLV), Las Vegas, USA; 3 Surgery, Kirk Kerkorian School of Medicine at the University of Nevada, Las Vegas (UNLV), Las Vegas, USA

**Keywords:** residency, residency applications, medical school curricula, general surgery, lic

## Abstract

Research statement

This study explores whether longitudinal integrated clerkship (LIC) students are competitive general surgery applicants and if they are perceived as adequately prepared for general surgery residency compared to traditional block rotation (BR) students.

Background/relevance of the study

There is increasing interest in LIC models of clinical education versus BR models. LIC students have been shown to perform similarly on examinations to BR students. However, while LICs seem well suited for students pursuing primary care specialties, little is known about how this approach impacts clinical education for surgery.

Design and methods

An electronic survey was prepared and approved by the Association of Program Directors in Surgery (APDS) and our university’s institutional review board (IRB). Ten multiple-choice questions were administered along with an option for narrative comments. Surveys were sent over a one-month period to members of APDS Listserv. Returned emails were de-identified, and the results were tabulated.

Results

From 43 responses, the majority identified as program directors (PDs) (65%) and reported being somewhat familiar or very familiar with LICs (90%). When asked about the statement “LIC students are prepared for surgical residency,” 22% “disagreed” or “strongly disagreed.” When asked “How would you rank a LIC prospective applicant in comparison to a BR student?” 35% responded that they would rank the LIC student lower or not at all. Of the respondents, 47% reported that they have current residents who were LIC students. Most of these residents (65%) are graded as “average” for current performance.

Conclusions

The results suggest that medical students who are trained using LICs may be disadvantaged when applying to general surgery residencies. Interpretation is limited by the small number of respondents, and it only reflects the opinions of active APDS Listserv members. Further study is needed to confirm these findings and elucidate the basis of perceived deficiencies in LICs. Students from these schools should be advised to obtain additional surgery experience.

## Introduction

In the United States, there are several models to educate medical students. Traditionally, medical schools use block rotations (BR) during the third-year clinical phase. Another model is the longitudinal integrated clerkship (LIC), which was created as an effort to restructure core clinical training to better support students’ learning and professional development [1]. Rural LIC models are beneficial in cities with lower clinical volumes so their students could maximize exposure to patients and medical specialties. The base idea of the LIC is that all clerkship specialties are completed simultaneously, so rather than the 4-6 weeks in one specialty (such as a traditional block), students will see and experience multiple specialties. Only a handful of medical schools in the country have adopted the LIC model, and they all have slight variations. The common core elements of LIC, as defined by the Consortium of Longitudinal Integrated Clerkship (CLIC), are the following: medical students participate in the comprehensive care of patients over time, have continuing learning relationships with these patients’ clinicians, and fulfill most of the year’s core clinical competencies across multiple disciplines concurrently [2].

Medical schools are continuously examining the best educational models to train their medical students. In the last decade, there has been much interest in longitudinal models of the third-year clerkship, especially in new medical schools, because some studies have shown that LIC students perform similarly or better on medical school board examinations compared to traditional block students [2-5]. Another reason to pursue LIC is that students in LIC curriculums study more broadly throughout their third year, which is theorized to increase foundational clinical knowledge and increase board scores [6]. Other studies have shown that LIC students perform better than their traditional block rotation peers in primary care residencies and are more likely to choose primary care specialties [7,8]. However, little research has been conducted to determine how LIC students perform compared to their peers in surgical residencies.

Thus, we chose to conduct this study to determine the perceptions of surgical residencies toward LIC students as general surgery applicants. The purpose of this study is to explore whether longitudinal integrated clerkship (LIC) students are competitive general surgery applicants and if they are perceived as adequately prepared for general surgery residency compared to traditional block rotation (BR) students.

## Materials and methods

For this study, an electronic survey was prepared and approved by the Association of Program Directors in Surgery (APDS) and the University of Nevada, Las Vegas, institutional review board (IRB). The survey contained 10 multiple-choice questions and an additional narrative comment section. The survey was sent to members of the Association of Program Directors in Surgery (APDS) on the Listserv mailing list. The survey was distributed electronically three times over a one-month period via the APDS Listserv Master. We received a total of 43 responses, and all responses were used for this study. The majority of participants identified as program directors (PDs); other identified roles included assistant program directors (APDs) and faculty members. Responses were de-identified, and the results were tabulated in an Excel (Microsoft® Corp., Redmond, WA) document. Percentages for each question answered were calculated to determine answer frequency.

Questions primarily focus on the participant’s role in their program, how familiar they were with LICs, if they have any residents who completed LIC curriculums, and how they perceive graduates from a LIC school to perform in a surgical residency. One of the survey questions is in regard to the American Board of Surgery In-Training Examination (ABSITE) performance. ABSITE is the in-training examination offered by the American Board of Surgery to general surgery residency programs. It is a formative assessment, consisting of multiple-choice questions, used by programs to measure residents’ progress in the application of surgical knowledge and the management of clinical problems related to surgery. The survey questions and answer choices are listed in Table 1.

**Table 1:** Survey questions with their answer choices ABSITE, American Board of Surgery In-Training Examination; LIC, longitudinal integrated clerkship

Question	Answer choices
1. Your role in your current program	Program director (PD), assistant program director (APD), or faculty member
2. Are you familiar with the LIC for undergraduate medical education?	Not familiar, somewhat familiar, or very familiar
3. Of the categorical residents currently in your program, how many have been trained in a LIC?	None, few, or several
4. How have LIC-trained residents performed in patient care outside the operating room?	No answer, below average, average, or above average
5. How have LIC-trained residents performed in the operating room?	No answer, below average, average, or above average
6. How have LIC-trained residents performed on the ABSITE as compared with traditional curriculum residents?	No answer, below average, average, or above average
7. Graduates from a LIC medical school are prepared for surgical residency	Strongly disagree, disagree, neutral, agree, or strongly agree
8. Graduates from a LIC medical school have strong surgical knowledge	Strongly disagree, disagree, neutral, agree, strongly agree
9. Graduates from a LIC medical school perform the same as those residents from a traditional medical school curriculum	Strongly disagree, disagree, neutral, agree, strongly agree
10. If all other qualifications were equal, how likely would you rank a student from a LIC school as compared to a student from a traditional school?	Not ranked, lower, same, or higher
11. Narrative comments	Free response

## Results

Of the 43 responses, 67% identified as program directors, while 7% identified as assistant program directors and 26% as faculty members. The majority of participants (91%) were familiar with LIC curriculums. A total of 47% of participants reported that they have current residents who graduated from LIC schools. Most of these residents (65%) are graded as “average” for their current performance in their residency program. When asked “Are LIC students prepared for general surgery residency?” 18% “disagreed” or “strongly disagreed,” 56% were “neutral,” and 26% ”agreed” or ”strongly agreed.” Lastly, when asked “How would you rank a LIC applicant in comparison to a classically trained applicant?” 30% responded that they would rank the LIC applicant lower or not at all, 63% would rank the LIC student the same, and 7% would rank the LIC student higher. All results are shown in Table 2.

**Table 2:** Tabulated results of survey responses LIC, longitudinal integrated clerkships; ABSITE, American Board of Surgery In-Training Examination; PD, program director; APD, assistant program director

Your role in your current program	Are you familiar with the LIC for undergraduate medical education?	Of the categorical residents currently in your program, how many have been trained in a LIC?	How have LIC-trained residents performed in patient care outside the operating room?	How have LIC-trained residents performed in the operating room?
PD: 29 (67%)	Not familiar: 4 (9.3%)	None: 23 (53.4%)	No answer: 23 (53.4%)	No answer: 23 (53.4%)
APD: 3 (7%)	Somewhat familiar: 21 (48.8%)	Few: 17 (39.5%)	Below average: 6 (13.9%)	Below average: 5 (11.6%)
Faculty: 11 (26%)	Very familiar: 18 (41.8%)	Several: 3 (6.9%)	Average: 13 (30.2%)	Average: 13 (30.2%)
			Above average: 1 (2.3%)	Above average: 2 (4.6%)
How have LIC-trained residents performed on the ABSITE as compared with traditional curriculum residents?	Graduates from a LIC medical school are prepared for surgical residency	Graduates from a LIC medical school have strong surgical knowledge	Graduates from a LIC medical school perform the same as those residents from a traditional medical school curriculum	If all other qualifications were equal, how likely would you rank a student from a LIC school as compared to a student from a traditional school?
No answer: 23 (53.4%)	Strong disagree: 3 (6.9%)	Strongly disagree: 2 (4.6%)	Strongly disagree: 3 (6.9%)	Not ranked: 3 (6.9%)
Below average: 5 (11.6%)	Disagree: 5 (11.6%)	Disagree: 6 (13.9%)	Disagree: 8 (18.6%)	Lower: 10 (23.2%)
Average: 14 (32.5%)	Neutral: 24 (55.8%)	Neutral: 27 (62.7%)	Neutral: 21 (48.8%)	Same: 27 (62.7%)
Above average: 1 (2.3%)	Agree: 5 (11.6%)	Agree: 7 (16.2%)	Agree: 7 (16.2%)	Higher: 3 (6.9%)
	Strongly agree: 6 (13.9%)	Strongly agree: 1 (2.3%)	Strongly agree: 4 (9.3%)	

Narrative comments left by participants are listed in Table 3. Overall, the comments were mixed between positive and negative perceptions. Several comments discussed if LIC students were interested in surgery and they would seek out extra surgical experiences outside of their LIC curriculums to be better prepared for residency; thus, the LIC did not affect the student’s performance. Some comments were blatantly negative suggesting that no LIC students are ready for surgical internships. One comment stated that their program consistently ranks LIC applicants lower and that they are clearly less competitive than classically instructed students. Lastly, a few comments brought up other reasons why they believe LIC students are less prepared outside of their longitudinal curriculum. They suggested that LIC students commonly are not exposed or as exposed to residents and medical structures during their clerkship, so they are unaware of expectations for resident performance. However, participants suggested that LIC students could remedy this by taking away rotations or visiting sub-internships at academic centers to experience what they are lacking in the LIC at their home intuitions.

**Table 3:** Narrative comment responses LIC, longitudinal integrated clerkship; subIs, sub-internships

Narrative comments
LIC students are ranked lower in the match process. For our program, they are clearly less competitive than their peers that are classically instructed.
In my experience, many students interested in surgery from a LIC have an extra drive to obtain the surgical experience. Those who have done so certainly have sought additional advanced surgical clerkships/subIs. I think performance would be the same ultimately.
Our new medical school is also LIC; the first class just started clinical rotations; so far, so good.
We have only had a few residents who came from longitudinal programs. I think that part of the issue is longitudinal rotations, and another part is that some of the students from longitudinal programs are not exposed to residents during their curriculum and so are unaware of expectations for resident performance. The curriculum can be disruptive to the clerkship if there is a combination of traditional clerkships with longitudinal requirements.
They are not ready for internship. They do catch up over time, but they start behind their peers.
The clinical surgical experience should include something akin to what the student will experience as a resident. When I query applicants about their clinical rotations and if they all have one-on-one experiences, I have found that they are poorly prepared for the team dynamics of a standard residency experience. Also, they tend to have unrealistic expectations of how busy they will be, how a busy team works, etc. If the LIC can assure that the student is not given a false sense of what residency and a real practice in surgery are like, then they will be fine.
I think the ultimate ranking of an applicant would have to do with letters from their core and senior surgical rotations regardless of their school’s curriculum.
It is important that LIC schools have an appropriate curriculum to help students prepare and choose surgery if desired, but 90% of their eventual abilities are due to their own efforts and taking advantages of the opportunities made available to them. Schools (LIC or traditional) also need to critically evaluate students well so not only are they motivated to succeed in medical school but more importantly so they are appropriately selected into programs where they can succeed.
LIC students interested in surgery graduate with better surgical skills because they end up connecting with a small cohort of surgeons who typically don’t teach and who enjoy having the same student. Students get a ton of attention and often end up doing more surgery than students at a traditional school where they are just another rotation. More surgery leads to more opportunity to operate, which leads to better starting skills. While they may have better early surgery skills, they are not better or not worse than any other, and the skill advantage disappears with time.
I would want to see some specific comments about their surgical experience and their performance in surgery specifically.
I am a convert. When our new medical school announced that they were going to use a LIC, I screamed bloody murder. I was so, so wrong. It may be designed to make primary care more attractive, but it creates great surgeons! The self-reliance around their education eliminates “educational entitlement,” the longitudinal relationships with patients truly teach accountability to the patient, and what’s not to like about having a resident who shows up and on day 1 can first assist, target with a laparoscope, tie knots, suture, and truly understand the lifestyle. My name is X, my cell is X, and I’ll be happy to speak to any surgeon who wants to listen.

## Discussion

The results of this study may suggest that medical students who are trained using LIC models may be disadvantaged when applying to general surgery residencies and that students from these schools should be advised to obtain additional surgery experiences that include interactions with residents and academic faculty. We believe that the key question to this study is number 10 (“If all other qualifications were equal, how likely would you rank a student from a LIC school as compared to a student from a traditional school?”) because this question will easily elucidate the perceptions of surgical residency PDs, APDs, and faculty. The answers to this question showed that the majority of participants would rank LIC students the same as traditional students, given that all other factors are the same. However, there were still 30% of participants that would rank the LIC students lower or not at all regardless of all other equal qualifications. This result could suggest that at some general surgery residency programs, LIC students will have a much more difficult chance to achieve admission into their program regardless of their qualifications.

After analyzing the results from this study, how can we change this negative perception of LIC students? Firstly, we can continue to read and study LIC students’ performance versus their peers. A study from the University of Minnesota, a school that uses a “rural LIC,” showed that their LIC students scored similarly on medical school board examinations and had the same surgical skills as their classically trained peers [5,9,10]. Additionally, a study from the University of North Dakota School of Medicine and Health Sciences showed that students from their rural LIC program, which requires surgery and obstetrics-gynecology rotations, received higher scores on clinical proficiency evaluations than their traditional peers [11]. Secondly, we can address other associated perceptions, such as LIC students having limited inpatient exposure, limited exposure to medical/hospital structure, limited exposure to residents, and unrealistic views of resident/physician workload. The belief that LIC students are inadequately prepared for internships is contrary to studies that have shown equal or better performance on objective evaluations for LIC students compared to traditional BR students during clerkships [12-14]. However, students should seek additional opportunities to remedy these perceived deficiencies and prove competency and understanding. Lastly, we can continue to advocate for LIC programs in rural settings because they help address the rural physician shortages by offering strong clinical experiences in rural settings to nurture student interest in future practice in such locations. A study found that introducing a LIC to a rural Australian community resulted in improved relationships with the community, fostered a culture of helping each other, and improved patient access to care [15].

This study had several limitations. Firstly, interpretation is limited by the small number of respondents. At this time (in early 2022), there are 258 general surgery residencies in the United States, so assuming that all of our 43 responses are from different programs, this study, at maximum, only represents the perceptions of 16.7% of general surgery programs. Secondly, this study only reflects the opinions of active APDS Listserv members. Thirdly, this study uses LIC as a broad term in the survey. There are several different LIC models used by medical schools in this country, and there can be substantial differences between the exposures and experiences of students from different programs and even students from the same program. Rural LIC students versus urban LIC students can also have a large difference in their exposures because of the populations they serve. Lastly, no statistical analysis was completed for this study. The questions were not asked in such a way to create a statistical model; therefore, this study’s significance is limited by only showing the subjective opinions of a small group of participants.

For the future directions of this study, further analysis is needed to confirm these findings and elucidate the basis of perceived deficiencies in LICs. We would like to increase the sample size to include more general surgery programs to get a better consensus on the perception of LIC students as general surgery applicants. Lastly, we think that it would be interesting to create a multi-institutional study with true LICs to determine students’ performance in general surgery residency versus their classically trained peers using in-service examination scores, resident evaluations, and skill competencies.

## Conclusions

This study is one of the first in the current literature to examine the perceptions of general surgery residency program directors toward LIC students. The results of this study suggest that some program directors perceive longitudinal integrated clerkship (LIC) students to have deficiencies in their medical education training and may not be adequately prepared for surgery residencies when compared to traditional block rotation (BR) students. Thus, medical students who are trained using LIC models may be disadvantaged when applying to general surgery residencies. Furthermore, it may be beneficial for students from LIC schools to obtain additional surgery experiences that include surgical sub-internships and additional interactions with residents and academic faculty. As the use of LIC models becomes more prevalent in medical education, it is important to continue studying the perceptions of LIC students compared to their peers. In the future, it would be beneficial to increase the sample size to better analyze the perceptions of the majority of program directors in order to determine how they view LIC students as general surgery applicants.
